# Immune checkpoint inhibitor-related endocrinopathies: from immune crosstalk to predictive biomarkers and precision management

**DOI:** 10.3389/fendo.2026.1935980

**Published:** 2026-09-16

**Authors:** Yang Liu, Tong Xu, Zhengyu Chen, Wenhui Zhang, Tao Wang, Weidong Yan, Yanpeng Xie, Haojing Li, Xiaojun Cai

**Affiliations:** 1 Department of Endocrinology, Heilongjiang Academy of Chinese Medicine, Harbin, China; 2 Department of Geriatrics, Heilongjiang Academy of Chinese Medicine, Harbin, China; 3 School of Heilongjiang University of Chinese Medicine, Harbin, China

**Keywords:** endocrine toxicities, immune checkpoint inhibitors, immune checkpoint pathways, immune-related adverse events, predictive biomarkers

## Abstract

Immune checkpoint inhibitors (ICIs) have significantly prolonged patient survival in cancer therapy; however, overactivated immune responses can lead to endocrine immune-related adverse events (irAEs), primarily manifesting as thyroid dysfunction, hypophysitis, adrenal insufficiency, and insulin-dependent diabetes mellitus. Some of these endocrine injuries are irreversible and lack early warning indicators. This narrative review summarizes current evidence regarding the mechanisms underlying ICI-induced endocrine toxicities, including immune checkpoint pathways, organ-specific autoimmune responses, host-related factors, predictive biomarkers, and emerging precision management strategies. It summarizes potential predictive biomarkers, including thyroid autoantibodies, cytokine profiles, and single-cell sequencing, and discusses emerging approaches, including imaging-based assessment, liquid biopsy, and multidimensional prediction models, while emphasizing their current limitations and the need for prospective validation. Finally, by integrating organ-specific mechanistic evidence, biomarker-based prediction, and risk-stratified monitoring, this review frames these advances within the concept of “proactive endocrine protection,” emphasizing the transition from passive toxicity management toward earlier identification and individualized surveillance.

## 1 Introduction

In the late 19th century, the intersection of immunology and oncology began to emerge. At that time, surgeon William Coley observed a phenomenon: after injecting inactivated bacteria into patients with sarcomas, the tumors showed signs of shrinking (1). In cancer treatment, the immune system is often one of the body systems affected. While conventional cytotoxic chemotherapy exerts antitumor effects, it frequently induces significant immunosuppression. Subsequently, targeted therapies gradually emerged, harnessing the recognition mechanisms of the innate immune system to direct immune cells toward specific attack on tumor cells (2). Over the past decade, immunotherapy has represented a milestone in transforming the treatment paradigms for both solid and hematologic malignancies. Owing to their broad applicability, monoclonal antibody drugs targeting immune checkpoint molecules—including CTLA-4, PD-1, and PD-L1—have prolonged survival in a large number of patients and even achieved sustained disease remission (3). ICIs restore immune function by blocking the relevant checkpoints; however, overstimulation of the immune response during treatment may lead to irAEs, which can be life-threatening in severe cases. Compared with the adverse reactions associated with chemotherapy and targeted therapy, the severity and timing of irAEs are considerably more unpredictable (4). Currently approved ICIs are primarily divided into two categories: one targets the CTLA-4 pathway, represented by ipilimumab; the other targets the PD-1/PD-L1 pathway, with commonly used drugs including atezolizumab, durvalumab, nivolumab, and pembrolizumab. The core mechanism of ICIs lies in blocking the binding of receptors to their ligands, thereby lifting the immunosuppressive state of T cells and enabling them to effectively recognize and kill tumor cells (5). Immune-related endocrine toxicity is largely irreversible; if not identified early, the risk of hospitalization and death is quite high. Unlike many other immune-related adverse events, endocrine toxicities frequently result in persistent hormonal dysfunction, requiring long-term monitoring and replacement therapy. The incidence and clinical phenotype of endocrine irAEs vary substantially according to the type of immune checkpoint targeted and treatment regimen. Despite increasing clinical recognition, the mechanisms underlying organ-specific endocrine injury remain incompletely understood, and reliable predictive biomarkers for early identification of high-risk patients are still lacking. Accordingly, this narrative review was structured according to the Population–Exposure–Outcome (PEO) framework. The overarching review question was: Among patients with cancer receiving ICIs, what is currently known about the clinical spectrum and underlying mechanisms of ICI-associated endocrine toxicities, and which biomarkers and emerging strategies may facilitate their early detection, risk stratification, and precision management? This review therefore synthesizes current evidence on the clinical characteristics, organ-specific mechanisms, host- and treatment-related susceptibility factors, predictive biomarkers, and emerging precision management strategies for ICI-associated endocrine toxicities.

## 2 Methodology

This narrative review was structured according to the Population–Exposure–Outcome (PEO) framework. The population of interest comprised patients with cancer receiving ICIs, the exposure was immune checkpoint blockade, including CTLA-4, PD-1/PD-L1, and other emerging checkpoint-targeted therapies, and the outcomes of interest were endocrine irAEs, particularly thyroid dysfunction, hypophysitis, adrenal insufficiency, and ICI-induced diabetes mellitus.

Relevant literature was identified through searches of PubMed supplemented by screening the reference lists of key reviews and relevant articles. The literature search was updated through August 2026, with no publication-date restriction. Search terms included combinations of “immune checkpoint inhibitor,” “PD-1,” “PD-L1,” “CTLA-4,” “endocrine toxicity,” “endocrine immune-related adverse events,” “thyroid dysfunction,” “hypophysitis,” “adrenal insufficiency,” “diabetes mellitus,” “mechanism,” “biomarker,” “prediction,” and related terms.

Eligible publications included clinical studies, systematic reviews and meta-analyses, relevant translational and mechanistic studies, and major clinical guidelines addressing the clinical characteristics, mechanisms, risk factors, predictive biomarkers, early detection, or management of ICI-associated endocrine toxicities. Case reports and preclinical studies were considered when they provided relevant evidence regarding rare endocrine toxicities or organ-specific mechanisms. Publications without relevant endocrine outcomes, studies unrelated to ICI therapy, conference abstracts lacking sufficient information, and duplicate publications were excluded.

Relevant information on endocrine toxicity type, clinical characteristics, proposed mechanisms, risk factors, biomarkers, and emerging detection or prediction strategies was extracted and organized thematically. Given the narrative nature of this review and the substantial heterogeneity in study designs, populations, endpoints, and mechanistic approaches, no formal meta-analysis or standardized risk-of-bias assessment was performed. Instead, greater interpretive weight was given to clinical guidelines, systematic reviews and meta-analyses, prospective studies, larger observational cohorts, and directly relevant mechanistic studies. The evidence was synthesized narratively according to endocrine organ and thematic domain, with particular attention to distinguishing relatively well-supported findings from preliminary, indirect, or extrapolated evidence. Accordingly, throughout the review, evidence was interpreted according to its source, including preclinical or mechanistic studies, clinical observational studies, retrospective cohorts, systematic reviews and meta-analyses, and consensus or guideline recommendations; findings derived from irAEs overall rather than endocrine-specific outcomes were explicitly identified as indirect or extrapolated evidence. A comparison of the present review with representative reviews and clinical guidelines on ICI-associated endocrinopathies is provided in **Table 1**.

**Table 1:** Comparison of the present review with representative reviews and clinical guidelines on ICI-associated endocrinopathies.

Reference (year)	Type	Primary focus	Biomarkers/prediction	Risk-stratified management	Organ-specific mechanistic evidence
Schneider et al. (2021) (5)	Guideline (ASCO)	Organ-specific diagnosis and management of ICI-related irAEs	Not a primary focus	Grade-based monitoring and management	Limited
Husebye et al. (2022) (32)	Guideline (ESE)	Evidence-based diagnosis, treatment, and follow-up of endocrine irAEs	Not a primary focus	Guideline-based monitoring and management	Limited
Wright et al. (2021) (13)	Narrative Review	Pathobiology, clinical manifestations, and treatment of endocrine toxicities	Limited	Limited	Yes
Iwama & Kobayashi (2025) (9)	Narrative Review	Clinical characteristics, management, biomarkers, and prognosis of endocrine irAEs	Yes	Discussed	Yes
Zhu et al. (2025) (30)	Consensus	Standardized definitions, evaluation, diagnosis, severity grading, and management of endocrine irAEs	Not a primary focus	Disease- and severity-based evaluation and management	Limited
Present Review (2026)	Narrative Review (PEO)	Integration of mechanisms, biomarkers, prediction, and precision management	Critical assessment of emerging biomarkers and prediction models	Integrated risk-stratified approach within “proactive endocrine protection”	Endocrine-specific evidence with evidence-strength distinction

## 3 Clinical spectrum and characteristics of ICI-induced endocrine toxicities

### 3.1 Thyroid dysfunction

Among ICI-related endocrine irAEs, thyroid dysfunction is the most prevalent. The overall incidence rates of ICI-associated hypothyroidism and hyperthyroidism are 6.6% and 2.9%, respectively, with considerable variation across different ICIs classes (6). Hyperthyroidism primarily results from destructive thyroiditis, which causes a massive passive release of preformed thyroid hormones into the circulation. In a minority of cases, however, it arises from Graves’ disease-like hyperthyroidism driven by autoantibodies that stimulate the TSH receptor. Notably, patients with a history of Graves’ disease are at significantly increased risk of developing hyperthyroidism following ICIs therapy, with prospective studies reporting an incidence as high as 15.8% (7). More than 50% of thyrotoxicosis cases eventually progress to hypothyroidism. Thyrotoxicosis is defined by suppressed TSH with concurrent elevation of FT4 and/or FT3, whereas primary hypothyroidism is defined by elevated TSH with reduced FT4 (8, 9). A positive TRAb result, when accompanied by clinical features of Graves’ disease, supports a diagnosis of Graves’ hyperthyroidism. However, transient and weakly positive TRAb may also occur during the acute phase of destructive thyroiditis, necessitating careful interpretation. The incidence of thyroid dysfunction varies substantially by ICIs regimen. Based on a pooled analysis (6), the respective incidence rates of hypothyroidism and hyperthyroidism were 3.8% and 1.7% for anti-CTLA-4 monotherapy, 7.0% and 3.2% for anti-PD-1 monotherapy, 3.9% and 0.6% for anti-PD-L1 monotherapy, and 13.2% and 8.0% for anti-PD-1/CTLA-4 combination therapy. Consistent with these findings, prospective cohort studies have reported overall thyroid irAE incidences (combining both hypo- and hyperthyroidism) of 9.9% for anti-PD-1 (10) 10.1% for anti-PD-L1 (11), and 37% for combination therapy (12).

### 3.2 Hypophysitis

Hypophysitis is the second most common ICI-related endocrine irAE. Unlike thyroid disorders, its diagnosis typically requires integration of clinical symptoms with laboratory findings and pituitary imaging, rather than reliance on any single modality.

For patients receiving ipilimumab (as monotherapy or in combination), the median time from treatment initiation to symptom onset is 9–12 weeks, whereas for those receiving anti-PD-1/PD-L1 therapy, this interval is approximately 26 weeks (13). Clinically, ICI-induced hypophysitis often presents with nonspecific symptoms related to pituitary hormone deficiencies, particularly secondary adrenal insufficiency, rather than symptoms of pituitary mass effect. In a cohort of 69 patients with ICI-induced hypophysitis, the most common presenting symptoms were fatigue (86%), headache (43%), and nausea and/or vomiting (39%), whereas hypotension (7%) and visual changes (4%) were less frequent (14). Secondary hypothyroidism and secondary hypogonadism are also frequently observed. Importantly, recovery differs across pituitary axes: ACTH deficiency is usually permanent and often requires lifelong glucocorticoid replacement, whereas thyroid and gonadal axis dysfunction may recover during follow-up. The incidence of hypophysitis varies substantially by ICI regimen, being highest with CTLA-4-based and combination therapy and relatively uncommon with PD-1/PD-L1 monotherapy (6, 14–16). Dual LAG-3/PD-1 blockade has also been associated with a higher incidence than PD-1 monotherapy (2.5% vs. 0.8%) (17, 18). Experimental studies further support an immune-mediated mechanism, demonstrating pituitary lymphocytic infiltration and circulating anti-pituitary autoantibodies following CTLA-4 blockade (19).

### 3.3 Adrenal insufficiency

Primary adrenal insufficiency (PAI) is a relatively rare but potentially life-threatening endocrine irAE associated with ICIs therapy (20). In differential diagnosis, the majority of ICI-related adrenal insufficiency cases (92.2%) are attributed to central adrenal insufficiency secondary to hypophysitis, whereas only 7.8% meet the criteria for primary autoimmune adrenalitis (21). The onset of ICI-related adrenal insufficiency typically occurs between the 2nd and 12th cycles of therapy, with a median time to onset of 4–6 months. Notably, 15.6% of cases present after ICIs discontinuation, with onset ranging from 2 weeks to 8 months post-treatment (21). Major clinical symptoms include fatigue (67.2%), nausea (28.1%), anorexia (25.0%), hypotension (21.9%), and altered mental status (21). These non-specific symptoms are often misattributed to tumor progression or other treatment-related toxicities. Severe cases may present with hyponatremia; hyperkalemia, when present, is more characteristic of primary adrenal insufficiency. The reported incidence of ICI-induced PAI is approximately 1.03% based on a large real-world pharmacovigilance database (22). ICI-related primary adrenal insufficiency is a rare endocrine immune-related adverse event, but it can be life-threatening and has been associated with serious clinical outcomes in pharmacovigilance reports (23). The pathogenesis of ICI-induced PAI remains incompletely understood but is thought to involve autoimmune-mediated adrenal destruction.

### 3.4 Insulin-dependent diabetes (T1DM)

ICI-DM is defined as severe and persistent insulin deficiency that develops following ICIs therapy. ICI-induced diabetes typically presents as sudden-onset hyperglycemia, resulting from rapid pancreatic β-cell destruction and consequent loss of insulin secretion, with a high risk of diabetic ketoacidosis (DKA) (24). Some patients initially diagnosed with type 2 diabetes (T2DM) may actually have latent autoimmune diabetes characterized by anti-GAD antibodies, a condition that increases their susceptibility to ICI-T1DM (25). ICI-related type 1 diabetes is a rare endocrine immune-related side effect, one large retrospective study reported an occurrence rate of 0.48% (25), although the reported incidence varies across populations and study designs (26). Unlike hypophysitis, which is predominantly associated with CTLA-4 blockade, ICI-DM occurs mainly in patients receiving PD-1/PD-L1 inhibitors, either as monotherapy or in combination with CTLA-4 inhibitors. Cases of ICI-DM following CTLA-4 inhibitor monotherapy are rare. Combination therapy targeting both PD-(L)1 and CTLA-4 pathways may further increase the risk of diabetes development compared with PD-1/PD-L1 inhibitor monotherapy, potentially reflecting greater disruption of immune tolerance (25). A proposed mechanism is that PD-1/PD-L1 blockade may facilitate the activation and expansion of autoreactive T cells, leading to pancreatic β-cell injury. Experimental studies, particularly in mouse models, support the involvement of both CD4^+^ and CD8^+^ autoreactive T cells in this process (27, 28). However, direct evidence establishing the pathogenic role and antigen specificity of autoreactive T cells in human ICI-DM remains limited. Mechanistic studies have demonstrated that PD-1 blockade can induce autoreactive T cells to release interferons (IFNs), which activate macrophages to kill β-cells via nitric oxide; however, histopathological findings in patients have shown predominant CD8+ T-cell infiltration with minimal macrophage involvement, suggesting that the relative contribution of these mechanisms may differ between the early effector phase and end-stage disease (26).

### 3.5 Severity assessment and clinical management of endocrine irAEs

Currently, the severity of endocrine irAEs is commonly assessed using the CTCAE grading system. However, this system primarily centers on the severity of endocrine symptoms and fails to adequately capture the fundamental clinical decision-making paradigm of endocrine irAEs, which is based on the binary assessment of hormone deficiency (29). Recently, expert consensus has attempted to establish more tailored grading criteria specifically for endocrine toxicities (30). In terms of clinical monitoring, thyroid function should be routinely assessed by measuring TSH, FT3, and FT4 prior to ICIs initiation and every 4–6 weeks thereafter (31). Morning cortisol and ACTH should be assessed promptly when symptoms or biochemical abnormalities raise suspicion of adrenal insufficiency; suspected hypophysitis warrants evaluation of cortisol, ACTH, TSH, and FT4, with additional pituitary testing and MRI as clinically indicated (32). For hyperglycemia management, a risk-stratified dynamic screening strategy is recommended: patients at low, intermediate, and high risk should undergo routine periodic blood glucose monitoring, post-glucocorticoid administration monitoring within 2 weeks followed by every 4 weeks thereafter, and daily self-monitoring using a glucose meter, respectively. Critical threshold values, such as blood glucose >15 mmol/L accompanied by ketone bodies >2 mmol/L, warrant immediate referral to emergency care (33). Multidisciplinary collaboration among oncologists, endocrinologists, and primary care physicians is emphasized in clinical management. Evidence indicates that endocrinologist-led care enables >80% of patients to receive guideline-recommended maintenance regimens, and the presence of E-irAEs generally does not justify ICI interruption (34). In most cases, ICI-associated thyrotoxicosis can be managed with observation or β-blockers for symptomatic relief; antithyroid drugs are rarely required, except in cases of Graves’ hyperthyroidism. Hypothyroidism should be treated with levothyroxine replacement (13). For adrenal insufficiency, physiological replacement with hydrocortisone (15–25 mg/day, divided into 2–3 doses) or equivalent prednisone is recommended, and patients should be educated on stress-dose doubling principles during infection, surgery, or other stressful events (35). Moreover, all patients with adrenal insufficiency must receive thorough education on stress-dose glucocorticoid administration and crisis self-management training (31). Collectively, these strategies establish a comprehensive management framework for endocrine irAEs founded on graded monitoring, reinforced by multidisciplinary collaboration, and centered on precise hormone replacement therapy.

## 4 Mechanistic insights into ICI-induced endocrine toxicities

### 4.1 Immune checkpoint pathways and ICI-induced endocrine autoimmunity

After establishing the clinical spectrum of endocrine irAEs, understanding their underlying immunological mechanisms is essential for explaining the organ specificity, timing, and variability of these toxicities. Immune checkpoint molecules maintain peripheral immune tolerance by transmitting inhibitory signals. CTLA-4 competitively binds to CD80/CD86 on antigen-presenting cells (APCs), thereby inhibiting T-cell activation and maintaining immune homeostasis by counteracting CD28-mediated positive co-stimulatory signals. PD-1 is widely expressed on the surfaces of immune cells such as T cells and B cells (36). Its natural ligand, PD-L1, is primarily found on tumor cells and tumor-infiltrating macrophages; the binding between PD-1 and PD-L1 induces and maintains immune tolerance (37). Activation of this pathway suppresses T-cell activation, promotes apoptosis of tumor-infiltrating lymphocytes, reduces the secretion of inflammatory cytokines, and enhances the release of IL-10 (38, 39). Similarly, LAG-3, another negative immune checkpoint, is expressed on the surface of T lymphocytes and negatively regulates the activation of memory T cells by interfering with the common activation pathway of CD4^+^ and CD8^+^ T cells (40). Clinical studies have confirmed that the combination of LAG-3 inhibitors and PD-1 inhibitors enhances the efficacy of anti-PD-1 therapy in advanced melanoma (17, 41).

However, while pharmacological blockade of the aforementioned inhibitory pathways enhances antitumor immunity, it also disrupts the body’s inherent immune homeostasis. Under physiological conditions, immune checkpoint pathways, particularly CTLA-4 and PD-1/PD-L1, contribute to the maintenance of peripheral immune tolerance by limiting excessive T-cell activation (13). Among these, CTLA-4 primarily acts during the T-cell activation phase within lymph nodes, while PD-1/PD-L1 limits the effector function of activated T cells in peripheral tissues (42); LAG-3 also participates in this network by negatively regulating T-cell proliferation (40). When ICIs block the aforementioned pathways, immune homeostasis may be disrupted, potentially triggering autoimmune processes that vary across different endocrine organs (13). In certain well-characterized ICI-induced endocrinopathies, particularly thyroiditis, activation and expansion of CD8^+^ cytotoxic T cells and CD4^+^ helper T cells have been observed (43). However, the relative contribution of these mechanisms varies among different endocrine organs and remains incompletely understood. Similar immune-cell infiltration patterns have been observed in ICI-induced hypophysitis. In a mouse model, anti-CTLA-4 antibody administration induced pituitary lymphocytic infiltration, complement activation, and circulating anti-pituitary autoantibodies (19). Importantly, autopsy studies in humans demonstrated inflammatory infiltration of the pituitary gland, predominantly composed of CD4^+^ T cells and B cells, providing direct pathological evidence of immune involvement in ICI-induced hypophysitis (44). However, the precise contribution of individual immune-cell subsets and their effector mechanisms remains incompletely understood. For other endocrine toxicities, including primary adrenal insufficiency and ICI-induced diabetes mellitus, the underlying immune mechanisms are less fully elucidated and may involve distinct effector pathways depending on the target organ and host factors (24). Studies have shown that clonally expanded CD8^+^ cytotoxic T cells are the primary infiltrating immune cells in thyroid tissue, and among them, the CD8^+^ effector T-cell subset expressing CXCR6, IFN-γ, and granzyme B plays a key role in thyroid injury (45). Lozano et al. (46) found that elevated activated CD4^+^ memory T cells and increased T-cell receptor diversity were associated with severe irAEs overall; however, their relevance to endocrine-specific irAEs remains unclear. Furthermore, T-cell activation and B-cell responses have been associated with the development of autoantibodies, including anti-thyroid peroxidase antibodies, anti-pituitary antibodies, and anti-insulin antibodies (47–50). However, whether T-cell clonal expansion directly contributes to pathogenic autoantibody production in endocrine irAEs remains uncertain and requires further investigation. In addition, the ectopic expression of immune checkpoint molecules in endocrine parenchymal cells is also an important mechanism—for example, the expression of CTLA-4 on pituitary hormone-secreting cells renders them directly susceptible to attack by anti-CTLA-4 antibodies, inducing a type II hypersensitivity reaction, which is pathologically characterized by complement deposition, lymphocytic infiltration, and typical hypophysitis (51). (**Figure 1**).

**Figure 1:**
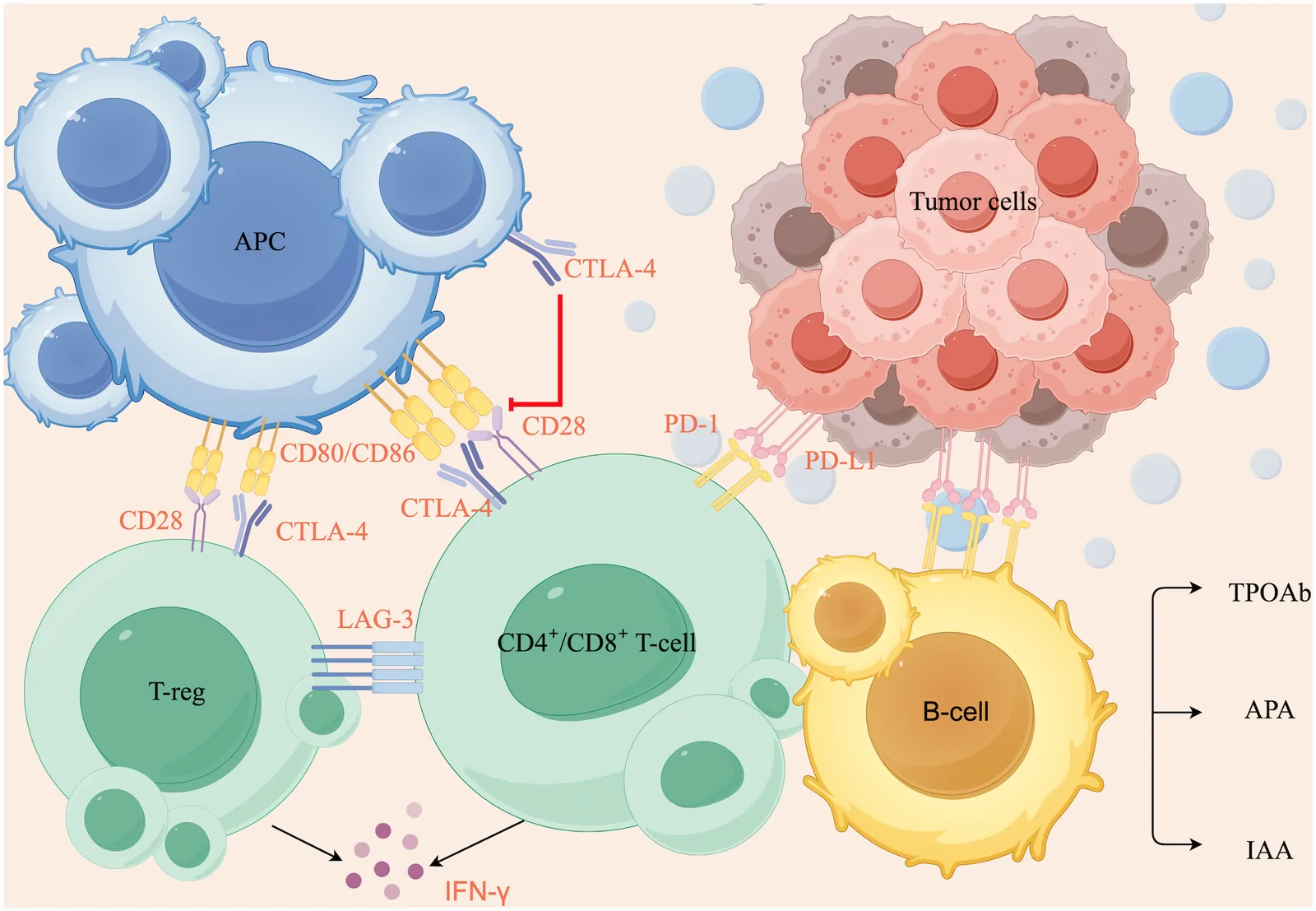
Overview of immune checkpoint pathways involved in ICI-associated endocrine autoimmunity. Under physiological conditions, inhibitory immune checkpoint pathways, including CTLA-4, PD-1/PD-L1, and LAG-3, contribute to the maintenance of peripheral immune tolerance. ICI-mediated blockade of these pathways may disrupt immune homeostasis and promote autoreactive immune responses involving T cells, B cells, autoantibodies, and inflammatory mediators, thereby contributing to endocrine tissue injury. The relative contribution of these immune mechanisms varies across endocrine organs and remains incompletely defined.

### 4.2 Organ-specific mechanisms

Mechanistic evidence for endocrine irAEs varies across organs, and some proposed mechanisms are extrapolated from studies of irAEs overall, animal models, or classical autoimmune diseases rather than directly demonstrated in endocrine irAEs. ICIs-induced endocrine toxicity is organ-specific and is closely associated with differences in immune molecule expression, tissue-specific antigens, and immune cell infiltration within target organs. These factors determine the presentation, severity, and progression of endocrine-related irAEs, providing clues for early diagnosis and targeted intervention. Thyrotoxicosis, for instance, is common during treatment with PD-1/PD-L1 inhibitors. Following ICI-mediated relief of T-cell suppression, autoreactive T cells may contribute to thyroid follicular injury (43), Thyroid tissue from patients with ICI-associated thyroiditis has shown infiltration by clonally expanded cytotoxic CD8^+^ T cells, including CXCR6^+^CD8^+^ effector cells characterized by increased IFN-γ and granzyme B expression (45). Clinically, ICI-associated thyroid dysfunction presents with heterogeneous patterns, including isolated hypothyroidism, transient thyrotoxicosis followed by hypothyroidism, and persistent hyperthyroidism. Destructive thyroiditis is the predominant pattern of ICI-associated thyroid injury, whereas Graves’ disease-like hyperthyroidism represents a less common antibody-mediated phenotype (6, 7). The risk of hypophysitis is substantially higher with CTLA-4 inhibitors, such as ipilimumab, than with PD-1/PD-L1 inhibitors, which may be partly explained by the expression of CTLA-4 within pituitary tissue. CTLA-4 inhibitors can directly bind to CTLA-4 expressed on pituitary cells, facilitating immune-mediated pituitary injury through mechanisms involving lymphocytic infiltration and cytotoxic T-cell activation (52). Following immune activation, lymphocyte infiltration into the pituitary may contribute to tissue injury through multiple mechanisms, including T-cell-mediated inflammation, autoantibody production, and complement activation. Although cytotoxic molecules such as perforin and granzyme have been implicated in immune-mediated tissue damage in other autoimmune disorders, their direct involvement in ICI-induced hypophysitis has not yet been demonstrated (53). Therefore, currently available evidence supports immune-cell infiltration and autoimmune-mediated injury as central features of ICI-induced hypophysitis, whereas the specific roles of CD8^+^ cytotoxic T cells and cytolytic pathways such as perforin/granzyme remain hypothetical and require further investigation. The pathogenesis of ICI-induced primary adrenal insufficiency (PAI) remains incompletely understood. Although ICI-induced PAI shares clinical features with autoimmune Addison’s disease, whether the underlying mechanisms are identical remains uncertain (54). In spontaneous autoimmune Addison’s disease, adrenal destruction involves autoreactive T cells, inflammatory cytokines, and autoantibody-mediated mechanisms, including anti-21-hydroxylase antibodies and complement activation (55). However, direct evidence demonstrating these immune pathways in ICI-induced PAI is currently limited. Therefore, ICI-induced PAI may involve overlapping but not yet fully defined mechanisms compared with autoimmune Addison’s disease (54). Case reports have described bilateral adrenal enlargement in some patients with ICI-induced PAI (56). Inflammatory responses during ICI therapy may also affect adrenal function; however, the mechanisms underlying adrenal dysfunction remain unclear. Clinical, steroid, and structural analyses suggest that normal ACTH levels alone may not exclude adrenal insufficiency during ICI therapy (57). Further studies are needed to clarify the underlying mechanisms. Currently, there is insufficient evidence regarding the specific molecular mechanisms by which ICIs directly interfere with this synthetic pathway, and further research is needed to elucidate these mechanisms. The pathogenesis of immune checkpoint inhibitor-induced diabetes (ICI-DM) is thought to involve immune-mediated destruction of pancreatic β-cells. Autoreactive T cells, particularly CD8^+^ cytotoxic T cells, have been proposed as key mediators; however, direct evidence establishing their pathogenic role and antigen specificity in human ICI-DM remains limited. In a subset of patients, classic islet autoantibodies—including glutamic acid decarboxylase 65 (GAD65) autoantibodies, insulinoma-associated protein 2 (IA-2) autoantibodies, insulin autoantibodies (IAA), and zinc transporter 8 (ZnT8) autoantibodies—are detectable (58, 59). Although autoantibody-mediated mechanisms, including complement activation, have been implicated in classical type 1 diabetes mellitus (60, 61), direct evidence demonstrating a pathogenic role for complement activation in ICI-DM remains limited. Therefore, current evidence suggests that ICI-DM is predominantly driven by T-cell-mediated cytotoxic mechanisms rather than established antibody-complement pathways. However, the potential contribution of humoral immune mechanisms requires further investigation. Unlike classic type 1 diabetes mellitus (T1DM), a significant proportion of ICI-DM patients are seronegative for islet autoantibodies (62).

Studies in non-obese diabetic (NOD) mice have demonstrated that both genetic knockout of PD-1 and pharmacological PD-1 blockade accelerate the onset of autoimmune diabetes. Both autoreactive CD4^+^ and CD8^+^ T cells respond to PD-1/PD-L1 blockade, promoting rapid disease progression. These experimental findings suggest that ICI-induced β-cell destruction may depend more heavily on direct T-cell-mediated cytotoxicity than on humoral immune mechanisms (63). Emerging evidence suggests that pancreatic injury in ICI-DM may involve both endocrine and exocrine compartments, with elevations in pancreatic enzymes and reductions in pancreatic volume reported in affected patients (64).

### 4.3 Host-related and treatment-related modulating factors

#### 4.3.1 Host genetic and demographic factors

The host genetic background represents an important intrinsic determinant of ICI-associated endocrine toxicity. HLA genotype polymorphisms influence individual susceptibility to ICI-induced autoimmune responses by modulating antigen presentation. Studies have shown that alleles such as HLA-A*26:01, DPA1*01:03, and DPB1*02:01 are significantly associated with the risk of ICI-induced thyroiditis (43). Notably, HLA-A*26:01 and DPB1*02:01 exhibit particularly strong associations with thyroid-related irAEs (43). ICI-associated thyroiditis shares genetic susceptibility loci with spontaneous autoimmune thyroid disease (AITD). Genome-wide association studies have identified multiple non-HLA susceptibility loci implicated in both conditions. Among these, PTPN22 encodes a lymphoid protein tyrosine phosphatase involved in the regulation of T-cell activation and immune tolerance. The R620W variant (rs2476601) has been associated with susceptibility to multiple autoimmune diseases, including type 1 diabetes and autoimmune thyroid diseases, although the strength of these associations varies across populations (65). Furthermore, Groha et al. (66)reported that single-nucleotide polymorphisms (SNPs) in the IL-7 gene are associated with an increased risk of irAEs. Patients carrying germline variants in IL-7 exhibited enhanced lymphocyte stability following ICIs treatment, suggesting that individuals with autoimmune-prone genetic backgrounds are more susceptible to excessive localized immune responses.

In addition to thyroid-related endocrine irAEs, specific genetic backgrounds have been implicated in other endocrine toxicities, particularly ICI-induced diabetes mellitus (ICI-DM). Several studies have demonstrated that HLA class II alleles, especially HLA-DR4 and HLA-DR3, are enriched among patients developing ICI-DM (59), suggesting that genetic susceptibility shared with classical type 1 diabetes mellitus may contribute to immune-mediated pancreatic β-cell destruction after immune checkpoint blockade. Beyond HLA polymorphisms, emerging evidence suggests that non-HLA genetic variants may also influence susceptibility to ICI-DM. A mutation in NLR family CARD domain containing 5 (NLRC5) has been reported in patients with ICI-DM, indicating that alterations in immune regulation and antigen presentation pathways may contribute to abnormal immune activation following ICI therapy (67). However, the functional significance of NLRC5 variants in ICI-DM requires further investigation in larger cohorts.

Genetic susceptibility has also been explored in ICI-induced hypophysitis. Studies suggest that specific HLA genetic backgrounds may increase the risk of developing ICI-associated pituitary dysfunction. Research by Yano et al (68). reported a potential association between HLA genotypes and ICI-induced hypophysitis; further studies by Kobayashi et al. (69) and Inaba et al. (70) identified associations with specific HLA alleles in patients who developed the condition, supporting the view that the host’s immunogenetic background may influence organ-specific autoimmune responses in the pituitary gland.

In addition to genetic susceptibility, sex may represent an important host-related factor influencing the risk and phenotype of endocrine irAEs. Sex-related differences have been reported for several ICI-associated endocrinopathies, although the direction and magnitude of these associations appear to vary according to the specific endocrine organ, ICI regimen, and underlying malignancy (71). Such differences may reflect interactions among sex hormones, sex-linked immune regulatory mechanisms, and baseline differences in autoimmune susceptibility. However, the available evidence remains heterogeneous, and sex should currently be considered a potential risk-modifying factor rather than an established independent predictor of endocrine irAEs (72).

#### 4.3.2 Treatment-related factors

The class and dose of ICIs are key treatment-related determinants of endocrine toxicity. Differences in the risk of endocrine adverse events among ICIs classes are primarily attributed to the distinct roles of CTLA-4 and PD-1/PD-L1 pathways in immune regulation (73, 74). A systematic review and meta-analysis by Yang et al. (75), including 26 randomized controlled trials and 13,824 patients, demonstrated distinct endocrine toxicity profiles across different classes of ICIs. PD-1 inhibitors were associated with increased risks of multiple endocrine adverse events, including hypothyroidism, hyperthyroidism, thyroiditis, hypophysitis/hypopituitarism, and type 1 diabetes mellitus, whereas CTLA-4 inhibitors showed particularly strong associations with hypophysitis/hypopituitarism. PD-L1 inhibitors were mainly associated with increased risks of thyroid dysfunction. These findings indicate that the pattern of endocrine toxicity differs according to the immune checkpoint targeted. The incidence of irAEs with anti-CTLA-4 antibodies, such as ipilimumab, exhibits a clear dose-dependent relationship. Clinical data indicate dose-dependent escalation of grade 3–4 irAEs, with incidence rates of 0%, 4%, and 8% at doses of 0.3 mg/kg, 3.0 mg/kg, and 10 mg/kg, respectively (76). Combination therapy with ICIs is an independent risk factor for hypophysitis and subsequent hypopituitarism (any grade), conferring a 4.3-fold higher risk compared with ICIs monotherapy. Furthermore, ICIs represent an independent risk factor for adrenal insufficiency (77).The combination of ICIs with other treatment modalities—including chemotherapy, targeted therapy, and local therapy—can synergistically increase the risk of endocrine irAEs through multiple mechanisms. Notably, Combination therapy with ICIs and tyrosine kinase inhibitors (TKIs) has received increasing attention because of its distinct endocrine toxicity profile. TKIs act on the tumor microenvironment by blocking targets such as VEGFR, PDGFR, and FGFR, thereby reducing tumor neoangiogenesis and tumor-associated immunotolerance, whereas ICIs, particularly anti-PD-1/PD-L1 antibodies, enhance antitumor immune responses. Although ICI–TKI combinations exhibit synergistic antitumor effects, they are associated with a higher incidence of endocrine adverse events than either ICI or TKI monotherapy (9.1% vs. 7.7% and 1.2%, respectively) (78).

#### 4.3.3 Gut microbiota and the immune microenvironment

The gut microbiota, frequently described as the “second human genome,” has been associated with host immune homeostasis and the occurrence of irAEs during ICI therapy. However, most available evidence concerns irAEs overall rather than endocrine-specific toxicities, and a causal relationship between gut microbial composition and endocrine irAEs has not been established (79, 80).

Preliminary evidence also suggests a potential association with endocrine irAEs. In a prospective metagenomic study, distinct gut microbial profiles were associated with organ-specific toxicities, including endocrine-system irAEs (81). Similarly, differences in intestinal microbiota composition have been observed in patients developing irAEs, including thyroid dysfunction, during anti-PD-1 therapy (82). However, these endocrine-specific findings remain preliminary, and larger prospective studies are needed to determine whether gut microbial profiles can reliably predict or contribute to the development of endocrine irAEs (83).

Beyond these observational associations, whether modulation of the gut microbiota can alter the risk of endocrine irAEs remains unclear. At the level of clinical intervention, Takada et al. (84) conducted a retrospective cohort study using inverse probability of treatment weighting (IPTW) in patients with non-small cell lung cancer receiving anti-PD-1 monoclonal antibodies (nivolumab or pembrolizumab). They reported that probiotic supplementation was associated with prolonged progression-free survival (PFS) and improved disease control rates. These findings suggest that exogenous microbiota intervention may modulate the host immune microenvironment and thereby influence ICIs treatment responses. However, whether such interventions specifically regulate the development of endocrine irAEs remains to be determined. (**Figure 2**).

**Figure 2:**
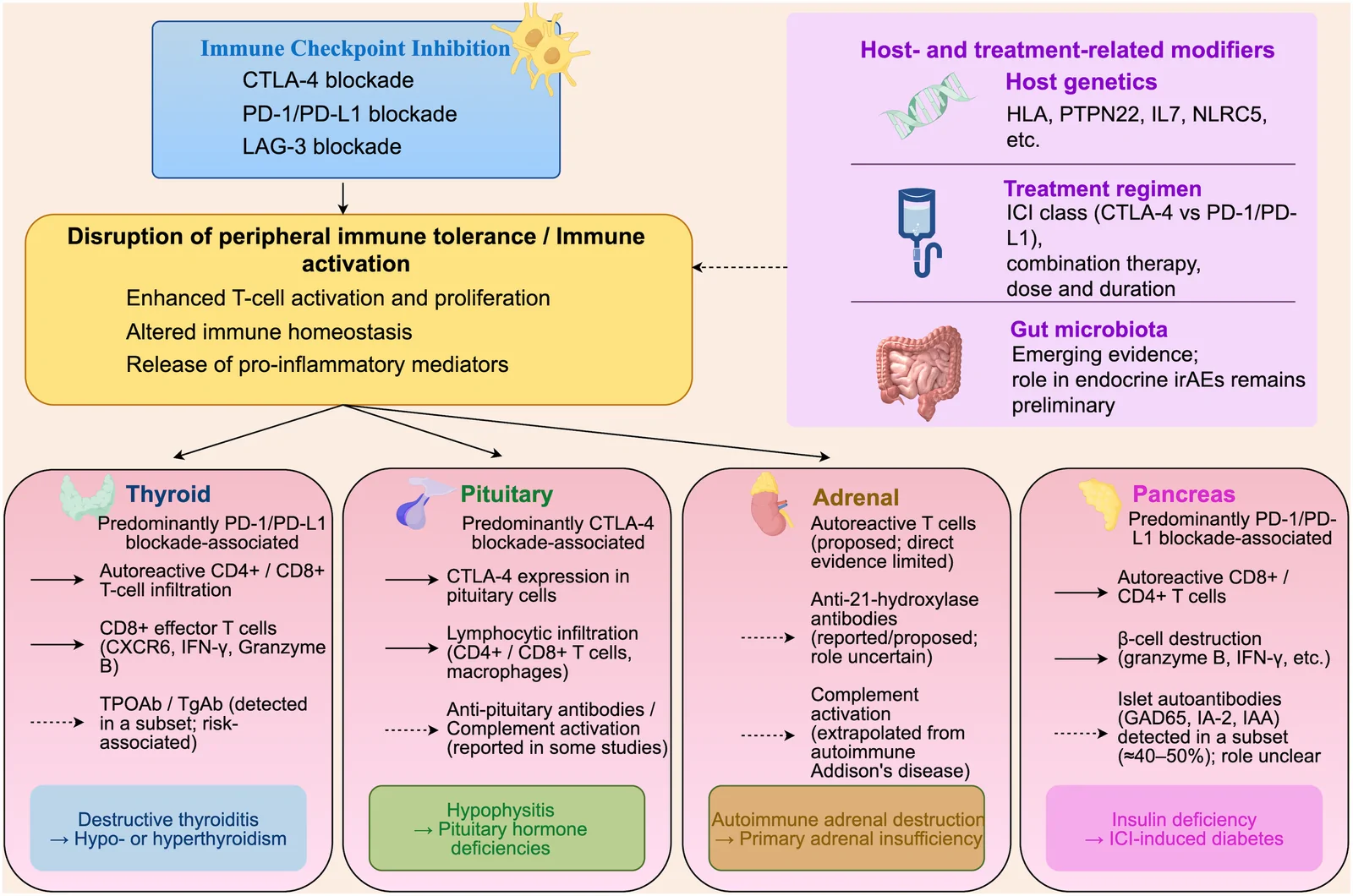
Organ-specific mechanisms underlying ICI-associated endocrine toxicities. Immune checkpoint blockade disrupts peripheral immune tolerance and promotes immune activation; however, the downstream mechanisms and strength of supporting evidence differ among endocrine organs. Solid arrows indicate mechanisms with relatively stronger evidence, whereas dashed arrows indicate proposed or incompletely established pathways. Host genetics, treatment regimens, and potentially the gut microbiota may further modify organ-specific susceptibility.

## 5 Predictive biomarkers and emerging strategies for early detection

Strategies for prediction and early detection of endocrine irAEs span a spectrum from established clinical monitoring to experimental technologies. In the following sections, we distinguish established clinical approaches from clinically accessible adjunctive markers and emerging investigational platforms, the latter of which generally require further prospective validation before routine clinical implementation.

### 5.1 Biomarkers for early prediction

Detectable thyroid autoantibodies at baseline or early during ICI therapy have been associated with an increased risk of thyroid irAEs. However, current evidence is insufficient to recommend routine thyroid autoantibody screening for all patients receiving ICIs. Instead, TPOAb and TgAb testing may be considered in selected patients with pre-existing autoimmune thyroid disease, abnormal baseline thyroid function, or increased risk of thyroid irAEs to assist risk stratification and follow-up. In the training cohort of a retrospective study involving 586 patients treated with PD-1/PD-L1 inhibitors, baseline TPOAb positivity (OR = 1.172, 95% CI: 1.110–1.237, P < 0.001) and TgAb positivity (OR = 1.105, 95% CI: 1.035–1.180, P = 0.003) were identified as independent predictors of thyroid dysfunction after adjustment for baseline TSH and platelet count (85). Prospective studies have confirmed that baseline positivity for thyroid autoantibodies is significantly associated with the risk of thyroid irAEs (P = 0.004) (86). Anti-pituitary antibodies have also been investigated as predictors of hypophysitis. Studies have shown that patients with ICI-induced isolated ACTH deficiency remain seropositive for anti-pituitary antibodies throughout treatment and follow-up (9), and that the presence of anti-GNAL and anti-ITM2B autoantibodies is significantly associated with hypophysitis (87). However, their current clinical utility remains limited because standardized anti-pituitary autoantibody assays are not routinely available. Furthermore, inflammatory mediators have emerged as potential biomarkers of immune-related toxicities, although evidence remains largely derived from studies of irAEs overall. Endocrine-specific evidence is currently strongest for thyroid dysfunction. In a prospective study of patients receiving ICIs, early changes in cytokine profiles, including increased IL-1β, IL-2, and GM-CSF levels, were associated with the development of thyroid irAEs, suggesting their potential utility for early risk stratification (88). However, most cytokine-based predictive models, including IL-7, IL-1RA, and CXCL13 composite scores, have been developed for irAEs overall rather than specific endocrine toxicities (89). Their predictive value for ICI-induced diabetes mellitus and hypophysitis remains to be established. More recently, Wu et al. identified pancreatic volume, islet autoantibodies, and immune-cell activation biomarkers as potential predictors of ICI-DM. Notably, integrating these parameters showed high predictive performance for ICI-DM (AUC = 0.968), supporting the potential value of multimodal biomarker-based risk stratification (90). Single-cell RNA sequencing has identified specific immune-cell subsets associated with irAEs. For example, exhausted CD8^+^ T cells interacting with IL-1β-expressing macrophages through chemokine–receptor interactions have been identified in PD-1 inhibitor-induced inflammatory arthritis (91). Although this study focused on joint toxicity, the identified macrophage–T-cell crosstalk provides mechanistic insights into irAEs more broadly. However, these findings should not be interpreted as validated biomarkers for endocrine irAEs. Whether similar cellular signatures occur in endocrine toxicities or provide predictive value for endocrine outcomes remains to be established in endocrine-specific prospective cohorts. Collectively, these biomarkers may support risk stratification for endocrine irAEs, but their applicability varies across endocrine organs. While some markers have endocrine-specific evidence, others are derived mainly from studies of irAEs overall and remain exploratory. Their clinical use is further limited by insufficient prospective validation and lack of standardized cutoff values.

### 5.2 Technological approaches for early detection

Early detection technologies encompass two main areas: routine laboratory tests and high-throughput technologies. Conventional laboratory testing forms the foundation: expert consensus recommends monitoring TSH and FT3/FT4 before treatment and every 4–6 weeks during the first 6 months after treatment initiation, followed by monitoring every 2–3 months thereafter (92). Patients with positive thyroid autoantibodies (TPOAb, TgAb) have a higher risk of developing thyroid irAEs (P = 0.004) (86). A study involving 264 patients confirmed that early-stage TPOAb (OR = 9.565) and TgAb (OR = 2.831) are predictors of thyroid dysfunction, and that their predictive performance appears superior to that of baseline markers (AUC: 0.655 vs. 0.571) (93). In studies of non-endocrine or mixed immune-related side effects, researchers have used single-cell RNA sequencing and found that certain immune cell groups increase in number after ICI treatment and are linked to these side effects (94, 95); whether these results also apply to endocrine side effects is still uncertain. Imaging may provide complementary information for the evaluation of endocrine irAEs in selected patients. Thyroid ultrasound can demonstrate structural changes associated with thyroiditis, including alterations in gland size, echogenicity, and vascularity; however, it is not recommended as a routine screening tool for all patients receiving ICIs. Thyroid function tests remain the cornerstone of thyroid irAE surveillance, whereas ultrasound may be considered when thyroid dysfunction is detected or thyroiditis is clinically suspected (96). A pituitary target-to-background ratio (TBR) cutoff of approximately 2.4 on 18F-FDG PET/CT, derived from ROC analysis, has been proposed as an early indicator of ICI-induced hypophysitis, with a sensitivity of 72.7% and a specificity of 90.9% (97). However, its clinical utility is constrained by limited availability of advanced modalities, undefined optimal timing, and transient radiological changes that can be missed or have already resolved at the time of imaging (98). Thus, pituitary imaging should be reserved for selected patients with atypical presentations or persisting symptoms despite adequate supplementation, and interpreted in conjunction with hormonal assessment, rather than serving as a routine screening tool (98).Liquid biopsy, as a non-invasive diagnostic method, holds considerable promise. Dynamic changes in circulating tumor DNA (ctDNA) levels have been associated with the development of irAEs overall (99). while plasma proteomic studies in NSCLC have identified baseline immune-related protein signatures associated with subsequent irAEs (100). Importantly, these findings have not been validated for the prediction of endocrine-specific outcomes. Therefore, ctDNA and plasma proteomic signatures should currently be regarded as hypothesis-generating approaches rather than established biomarkers for endocrine irAEs. Endocrine-specific prospective validation is required before their incorporation into clinical risk-stratification strategies.

### 5.3 Prediction models and risk stratification

In studies of immune-related side effects, prediction models that combine multiple factors now often include patient baseline details, treatment information, and biomarker patterns; but evidence specifically about endocrine problems is still more limited (101–103). In the field of thyroid toxicity prediction, nomogram-based models have demonstrated promising results. A study of 586 patients demonstrated that baseline TSH, TgAb, TPOAb, and platelet count were independent predictors; the model showed good discrimination in both the training set (AUC = 0.863) and validation set (AUC = 0.885), although calibration metrics were not reported (85). A meta-analysis identified combination ICIs therapy (OR = 3.26, 95% CI: 2.22–4.79), ICIs combined with targeted therapy (OR = 2.71, 95% CI: 2.11–3.47), elevated baseline TSH (OR = 1.30, 95% CI: 1.10–1.53), as well as positive TgAb (OR = 14.23, P < 0.001) and TPOAb (OR = 3.75, P < 0.001), as independent risk factors for endocrine irAEs (E-irAEs) (102). Separately, a recent study developed predictive models using 11 machine learning algorithms; the LightGBM model achieved the best performance (internal AUC = 0.96; external AUC = 0.71), highlighting a substantial drop in generalizability that warrants cautious interpretation (104). Risk stratification based on these models may help identify patients requiring closer endocrine surveillance, including more frequent assessment of thyroid function, glucose, and pituitary–adrenal function. For patients identified as high risk, more frequent monitoring may be warranted, although the optimal interval remains to be defined in prospective trials. Following ICIs initiation, a tiered management strategy should be implemented following diagnosis and severity assessment. Despite promising discrimination in some studies, current prediction models remain limited by small or retrospective cohorts, incomplete feature sets, insufficient external validation, and reduced generalizability; therefore, their reported performance should be interpreted cautiously before application to routine endocrine risk stratification (105).

## 6 Future perspectives and unmet needs

Although progress has been made in the management of ICI-related endocrinopathies, several limitations should be considered when interpreting current findings, including differences in tumor types, treatment regimens, and outcome definitions, as well as possible pharmacovigilance and publication biases. Some reported associations may also be influenced by underlying autoimmune susceptibility, cancer-related inflammation, or treatment-related immune activation, rather than a direct causal relationship; therefore, further research is needed to address these limitations and improve mechanistic understanding, risk prediction, and clinical management. Future research should systematically advance along three interconnected directions. At the mechanistic level, it is essential to elucidate the molecular determinants of organ-specific injury, including the recognition of tissue-restricted autoantigens, the interplay among the gut microbiota, immune system, and endocrine axis, as well as the irreversible molecular cascades leading to permanent destruction of pancreatic β-cells or the adrenal cortex, thereby laying the foundation for rational target discovery. In terms of prediction, multi-omics features should be integrated into high-dimensional biomarker panels, combined with risk modeling that incorporates dynamic biomarker trajectories during treatment, to enable pre-identification of high-risk patients; concurrently, the potential clinical utility of non-invasive technologies such as liquid biopsy and wearable endocrine monitoring devices should be evaluated in prospective endocrine-specific studies. On the therapeutic front, the development of selective immunomodulatory agents that restore immune tolerance without compromising antitumor efficacy—such as biased Treg biologics or tissue-targeted nanodelivery systems—represents a paradigm shift from nonspecific management approaches toward precision endocrine care, emphasizing early identification of hormone deficiencies, individualized hormone replacement, and mechanism-informed monitoring strategies. Furthermore, preventive strategies for genetically or serologically high-risk populations, as well as pharmacogenomics-guided individualization of hormone replacement regimens, hold promise for further optimizing the risk–benefit balance of ICI therapy. Collectively, integrating organ-specific mechanistic evidence, dynamic prediction, and risk-stratified monitoring provides a unified framework for transitioning from passive toxicity management to proactive endocrine protection.

## 7 Conclusion

ICI-associated endocrinopathies are important irAEs and mainly involve the thyroid, pituitary, adrenal glands, and pancreatic β-cells; some of these endocrine disorders may lead to long-term hormone deficiency. Current studies show that the clinical features and possible mechanisms are different among endocrine organs, and the available evidence is also not equally strong for each type of endocrine toxicity. At present, routine biochemical and hormone testing remains the main method for clinical monitoring and early detection, while thyroid autoantibodies may provide additional information for risk assessment in selected patients. Other potential biomarkers and methods, including cytokine profiles, advanced imaging, liquid biopsy, multi-omics approaches, and prediction models, have shown possible value for earlier identification of endocrine irAEs; however, most of these methods still lack sufficient prospective and endocrine-specific validation, so they are not yet suitable for routine clinical use. Therefore, current clinical practice should continue to focus on baseline assessment, regular endocrine monitoring, early identification of hormone deficiency, and appropriate hormone replacement; with further evidence, these emerging biomarkers and prediction methods may provide additional support for risk assessment and individualized monitoring.
